# Microcephaly type 22 and autism spectrum disorder: A case report and review of literature

**DOI:** 10.1080/19585969.2024.2359918

**Published:** 2024-06-03

**Authors:** Jiqiang Ma, Yu’e Liu, Kaijun Zhao

**Affiliations:** Department of Neurosurgery, Shanghai East Hospital, School of Medicine, Tongji University, Shanghai, China

**Keywords:** Autism Spectrum Disorder, cryptogenic vascular dissection, stent neurodevelopmental disorder

## Abstract

**Introduction:**

Autism Spectrum Disorder (ASD) is a complex neurodevelopmental disorder with a multifaceted etiology. This case report explores the ischemic cryptogenic vascular dissection as a potential underlying cause of ASD.

**Methods:**

A 9-year-old child presented with symptoms of ASD, including social interaction difficulties, repetitive behaviors, and cognitive challenges. Despite conventional ASD treatments, significant improvement was only observed after addressing an underlying ischemic cryptogenic vascular dissection identified through DCE-CT.

**Results:**

Following a reconstructive treatment approach to the vascular dissection, the patient showed marked improvement in cognitive functions, social abilities, and a reduction in ASD-related symptoms whether during the perioperative period or during approximately 5-month follow-up.

**Conclusion:**

This case suggests that ischemic cryptogenic vascular dissection may contribute to the symptoms of ASD. Identifying and treating underlying vascular anomalies may offer a new avenue for mitigating ASD symptoms, emphasizing the need for comprehensive diagnostic estimations in ASD management.

The main clinical manifestations of Autosomal Recessive Primary Microcephaly Type 22 include microcephaly, short stature, and developmental delay, among others. The NCAPD3 gene, located on chromosome 11, is one of the pathogenic genes for this condition. In the sequencing data of the family of the child in this case, heterozygous variations consistent with the condition were detected in the NCAPD3 gene. The child exhibited a range of neurodevelopmental disorders (Table 1) and was also diagnosed with Autism Spectrum Disorder (ASD, ADOS-2). Herein, the causation and treatment of the child’s genetic mutations will be further discussed.

**Table 1.** Patient symptoms, onset time, and changes before and after intervention.

Symptoms	Onset time	Before intervention	After intervention (0–5M)			
			Worsened	No change	Improvement	Significant improvement
Sleep disorders	3 M	NR				**+**
Dietary stereotypes	6 M	GW				**+**
Strabismus	10 M	GW			**+**	
Cold extremities	1 Y	NR				**+**
Drooling	1 Y	NR			**+**	
Speech disorders	1.5 Y	NR				**+**
Memory impairment	1.5 Y	NR				**+**
Social interaction difficulties	1.5 Y	GW			**+**	
Cognitive challenges	2 Y	GW				**+**
Poor mathematical ability	2 Y	NR				**+**
Rigid body	2 Y	NR				**+**
Chewing disorders	2 Y	NR				**+**
Repetitive behaviours	2.5 Y	GW			**+**	
Strange taste buds	2.5 Y	NR			**+**	
Self-control disorder	3 Y	NR			**+**	
Stereotypic behaviour	3 Y	GW			**+**	
Hand weakness	3 Y	NR				**+**
Dysfunction in coordination of limbs	3.5 Y	GW			**+**	
Gastrointestinal dysfunction	4 Y	NR				**+**
Auditory abnormality	4 Y	NR			**+**	
Emotional control disorder	7 Y	NR				**+**
Dizziness and vertigo	7 Y	NR				**+**

*Note:* M: months; Y: years; NR: no relief; GW: gradually worsening.

## Case presentation

We report the case of a nine-year-old child presenting initially with disturbed sleep patterns (Dawson et al. 2023), inadequate dietary intake (Dawson et al. 2023), strabismus, cold extremities and drooling which emerged at the age of one year or younger. These symptoms were later accompanied by repetitive behaviours, muscular weakness, social interaction difficulties, cognitive challenges, dizziness, vertigo, speech disorders, and limb weakness, particularly affecting the distal ends of the upper limbs, thereby hindering the ability to manipulate objects such as door handles and locks (Table 1). Despite extensive evaluations including fundus examination, electroencephalogram, cranial MRI, and MRA, the aetiology remained elusive. At the age of 4, this child was diagnosed as Autism Spectrum Disorder (ASD) based on Autism Diagnostic Observation Schedule-2(ADOS-2, Module 1), and the multiple heterozygous variations of the NCAPD3 gene (chr11:134022951/Paternal, NM-015261:exon35:c.4389-4C > G;chr11:134051019/Maternal, NM-015261:exon20:c.2512A > G[p.I838V]) were detected in the sequencing data of the affected family. The child received treatments aligned with ASD protocols (Lord et al. 2018; Hirota and King 2023) and psychosocial interventions (Lord et al. 2020; Wood et al. 2020), which yielded unsatisfactory results over five years. Moreover, intervention with antiplatelet drugs was minimally effective in addressing cold extremities.

## Diagnosis of ischaemic cryptogenic vascular dissection

**Figure 1.**
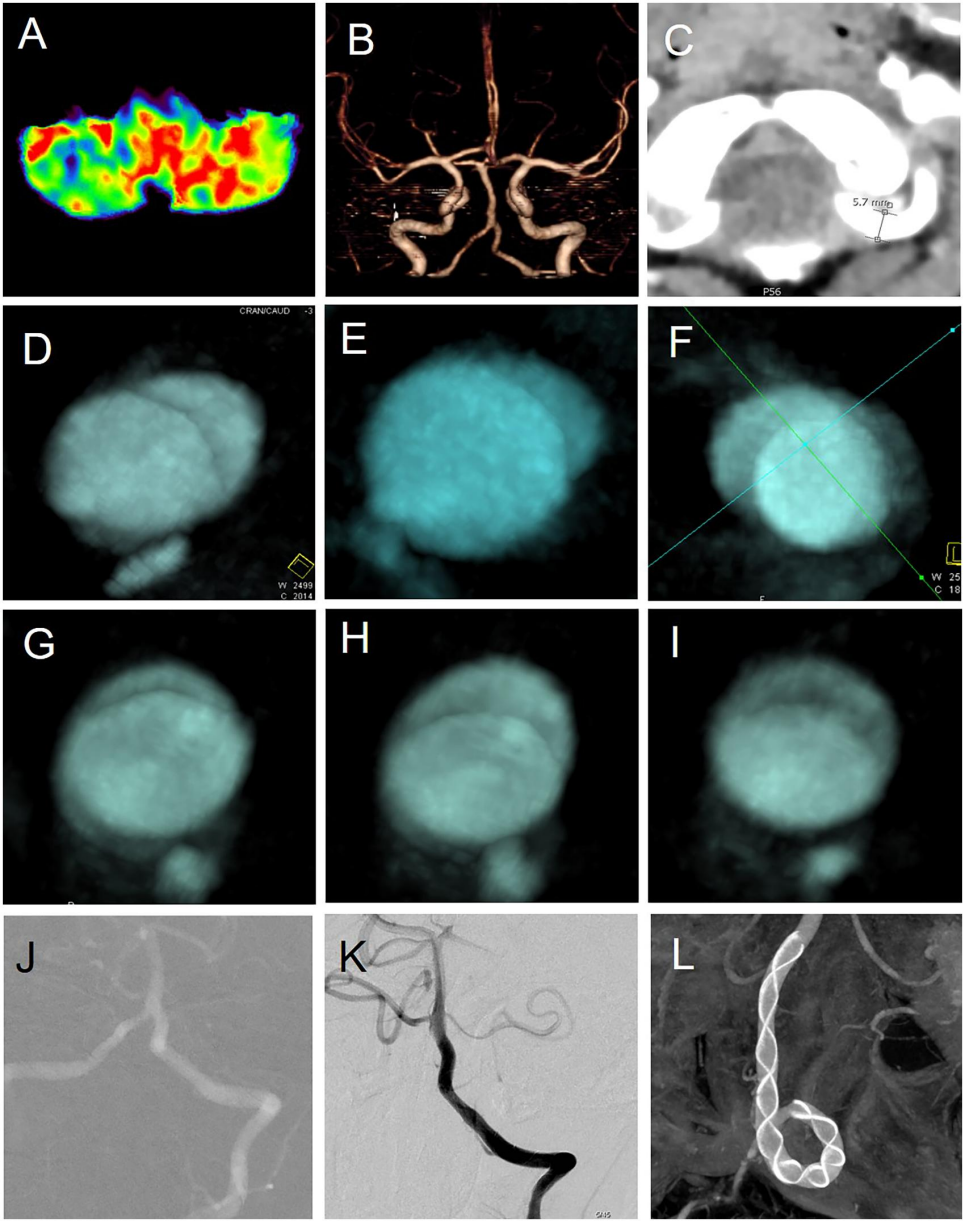
Treatment of cryptogenic vascular dissection related to neurodevelopmental disorders presenting with autism spectrum disorder. Hypoperfusion in the left cerebellar hemisphere (A), but with normal intracranial vertebral artery morphology (B). The vertebral artery dilates at the entry site, with a diameter of 5.7mm (C); two sequential DCE-CTs showed progressive morphological changes over different periods at the same location (D–F vs G–I); (J–L) the left cryptogenic vascular dissection was reconstructed using a stent.

Significant ischaemic hypoperfusion was identified in the left cerebellar hemisphere and portions of the brainstem *via* CT perfusion imaging (Figure 1(A)). Despite normal intracranial vascular morphology on DSA and CTA (Figure 1), Dynamic contrast-enhanced computed tomography (DCE-CT) revealed a chronic, long-segment vascular dissection in the left intracranial vertebral artery, with progressive morphological changes over a three-day period (Figure 1(D–F) vs Figure 1(G–I)). This finding led to the diagnosis of ischaemic cryptogenic vascular dissection, a condition challenging to detect through routine diagnostic processes. Here, we term it as ‘the cryptogenic vascular dissection’ for the concealment of lesions within normal vascular morphology.

## Treatment and follow-up of ischaemic cryptogenic vascular dissection

The child began premedication with dual antiplatelet therapy, consisting of 75 mg of clopidogrel and 100 mg of aspirin, at least three days prior to the procedure. Systemic intravenous heparin was administered to achieve an activated clotting time between 250 and 300 s when the femoral sheath was placed. Reconstructive treatment of the left entire dissected segment was performed using a 5 mm × 45 mm Tubridge Flow Diverter, extending at least 5 mm beyond the border of the dissecting segment of the parent artery. Post-procedure, the child was maintained on a dual antiplatelet regimen for six weeks, comprising daily doses of 75 mg clopidogrel and 100 mg aspirin. This was followed by 37.5 mg of clopidogrel and 50 mg of aspirin for 4.5 months, and subsequently, indefinite monotherapy with 25 mg of aspirin. During the perioperative period and approximately five months of follow-up after treatment of this left dissection, multiple systemic symptoms of the patient improved or significantly improved (Table 1), and no preoperative symptoms worsened.

## Discussion

The clinical manifestations of this child are consistent with ASD and partially related to the phenotype of Type 22 primary microcephaly caused by mutations in the NCAPD3 gene. The sequencing data from the family revealed the variant c.4389-4C > G from the father and c.2512A > G (p.I838V) from the mother. In this case, the mutation is rare and of unclear clinical significance.

ASD is characterised by a spectrum of complex, lifelong neurodevelopmental disabilities impacting communication and socialisation, alongside repetitive behaviours. In this case, despite normal vertebral artery morphology, the identification of hypoperfusion and a chronic vascular dissection suggests a novel aetiological pathway for ASD symptoms through cerebral hypoperfusion, which may be symmetrical or asymmetrical.

Following a reconstructive treatment approach to the dissection (Figure 1(J–L)), the patient exhibited significant improvements in symptoms previously attributed to ASD, including social interaction, strabismus, ocular motility, language skills, the ability to swallow and chew, distal upper limb strength, memory, sleep quality, dietary preferences, gastrointestinal function, emotional stability, and extremity temperature regulation, and so on (Table 1). This suggests that ischaemic cryptogenic vascular dissection, though difficult to detect, may play a role in the aetiology of ASD and that addressing this underlying condition could substantially mitigate ASD symptoms, which also supports that ASD is a neurovascular disease (Wang et al. 2023).

Overall, the discovery of highly concealed ischaemic vascular dissection in this child, which significantly improved a range of neurodevelopmental disorders after the dissection was relieved, suggests that these so-called pathogenic gene mutations may be induced by a long-term ischaemic environment or could be false positives due to the limitations of high-throughput sequencing technology. Further confirmation with sanger sequencing may be necessary when required in more data in future.

## Conclusion

Identifying and treating underlying cryptogenic vascular dissection may offer a new avenue for mitigating ASD symptoms, highlighting the need for comprehensive diagnostic evaluations in ASD management.
